# Leiomyosarcoma of the transverse colon with lymph node metastasis and malignant transformation: a case report

**DOI:** 10.1186/s40792-020-00998-4

**Published:** 2020-10-02

**Authors:** Tomoya Tago, Shuji Suzuki, Jun Kuroda, Ryutaro Udou, Kiyotaka Nishida, Yukio Oshiro, Jirou Shimazaki, Keni Kou, Yukio Morishita, Mitsugi Shimoda

**Affiliations:** 1 Department of Gastrointestinal Surgery, Tokyo Medical University Ibaraki Medical Center, 3-20-1 Chuo, AmimachiIbaraki, Inashiki-gun 300-0395 Japan; 2 Department of Pathology, Tokyo Medical University Ibaraki Medical Center, 3-20-1 Chuo, AmimachiIbaraki, Inashiki-gun 300-0395 Japan

**Keywords:** Leiomyosarcoma, Colorectum, Lymph node metastasis, Malignant transformation

## Abstract

**Background:**

The concept of GIST was established in 1998, clearly differentiating between gastrointestinal leiomyosarcoma and GISTs among gastrointestinal mesenchymal tumors. Lymph node metastasis is extremely rare in true gastrointestinal leiomyosarcoma, and there are no reports of malignant transformation from leiomyoma.

**Case presentation:**

The patient was an old woman who had undergone endoscopic mucosal resection for an Is polyp on the left side of the transverse colon at the age of 73. She was diagnosed with leiomyoma with positive surgical margins. Subsequently, she presented to our institution with a sensation of pressure in the upper abdominal region as a chief complaint at the age of 76 years. Abdominal computed tomography and colorectal endoscopy showed a tumor lesion with invagination of the intestines in the transverse colon, the same site as that of the previously resected leiomyoma. A biopsy suggested a smooth muscle tumor, and we performed partial left transverse colectomy and lymph node dissection under a diagnosis of recurrence and enlargement of the previously incompletely resected leiomyoma. Histopathological examination revealed spindle-shaped tumor cells, and the mitotic activity was 30–40/10 high-power field. Tumor cells were immunohistologically positive for α-smooth muscle actin and h-caldesmon; partially positive for desmin; negative for c-kit, CD34, DOG-1, and the S-100 protein; and showed a Ki-67 labeling index of 70–80%. She was diagnosed with leiomyosarcoma malignantly transformed from leiomyoma. Metastasis was found in 1 of the 14 resected lymph nodes. The patient did not undergo adjuvant chemotherapy, but has survived with no recurrence at 2 years after the surgery.

**Conclusions:**

We have reported a case of leiomyosarcoma of the transverse colon with lymph node metastasis that was malignantly transformed from a leiomyoma.

## Background

Since discovery of the c-kit gene mutation by Hirota et al. in 1998 [1], gastrointestinal leiomyosarcomas have been recognized as distinct from gastrointestinal stromal tumors (GISTs), as opposed to their earlier classification as GISTs [2–4]. True gastrointestinal leiomyosarcomas comprise less than 0.1–10% of all gastrointestinal mesenchymal tumors, occurring least frequently in the stomach and most frequently in the large intestine [5]. Lymph node metastasis is extremely rare in true gastrointestinal leiomyosarcoma, and there are no reports of malignant transformation in leiomyoma of the colon.

## Case presentation

**Fig. 1:**
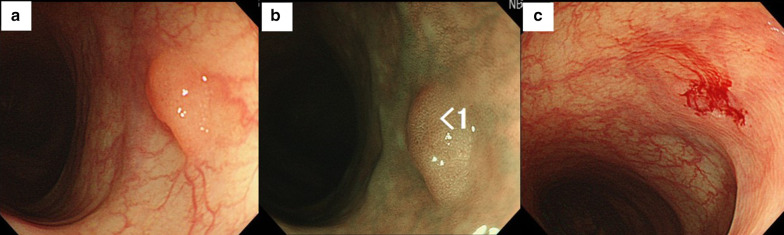
Colorectal endoscopy findings. **a** A type 0-Is polyp (5 mm) is observed in the left transverse colon. **b** Narrow band imaging findings **c** Resected surface after endoscopic mucosal resection

**Fig. 2:**
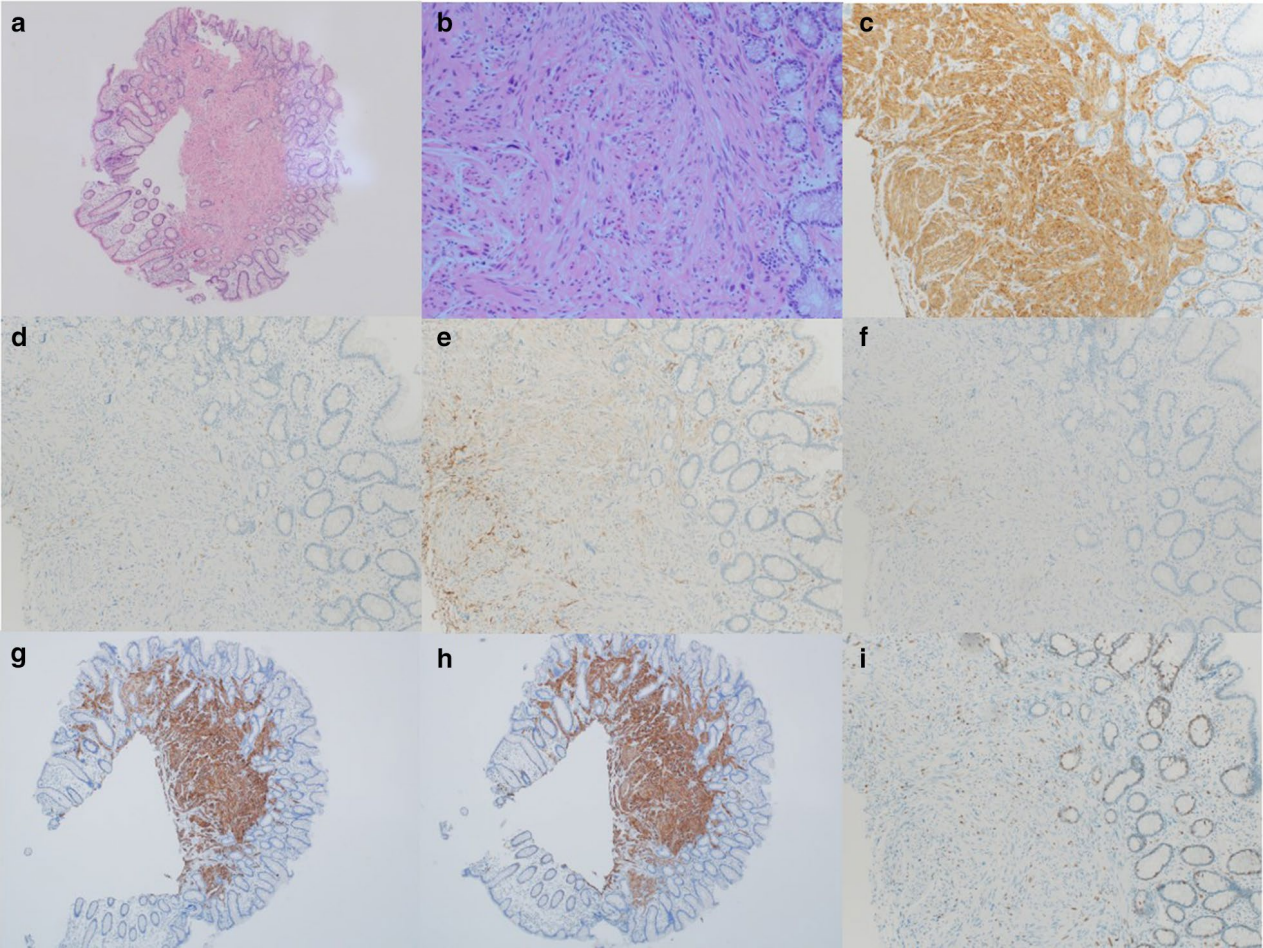
Microscopic and immunohistological findings. **a** Hematoxylin–eosin staining (×40). Spindle-cell proliferation with acidophilic endoplasmic reticula was observed in the deep portion to the inside of the specimen’s mucosa, and the resected margin was positive. **b** Hematoxylin–eosin staining (×200). Cells with slight nuclear atypia were found, but there was no clear mitotic activity. **c** Positive for α-smooth muscle actin (×100). **d** Negative for c-kit (×100). **e** Negative for CD34 (×100) **f** Negative for the S-100 protein (×100). **g** Positive for desmin (×40). **h** Positive for h-caldesmon (×40). **i** Ki-67 labeling shows a few positive cells

The patient was an old woman who had undergone endoscopic mucosal resection (EMR) for an Is polyp (sessile type: 5 mm) on the left side of the transverse colon at the age of 73 years (Fig. 1a–c). Total colonoscopy was performed at the time, showing that the polyp was located 50 cm from the anal verge and observing no other lesions. Hematoxylin–eosin (HE) staining of the specimen showed proliferating spindle cells from the deep portion to the inside of the mucosa, with positive surgical margins (Fig. 2a). Some spindle cells had slight nuclear atypia, but mitotic activity was not clearly observed (Fig. 2b). Tumor cells were immunohistologically positive for α-smooth muscle actin (α-SMA) and negative for c-kit, CD34, and the S100 protein. Accordingly, the polyp was diagnosed as a leiomyoma (Fig. 2c–f). The patient did not return to the outpatient clinic after the discharge to receive her pathological results and did not visit the hospital again for 3 years, meaning that she did not keep to our follow-up and treatment protocols.

**Fig. 3:**
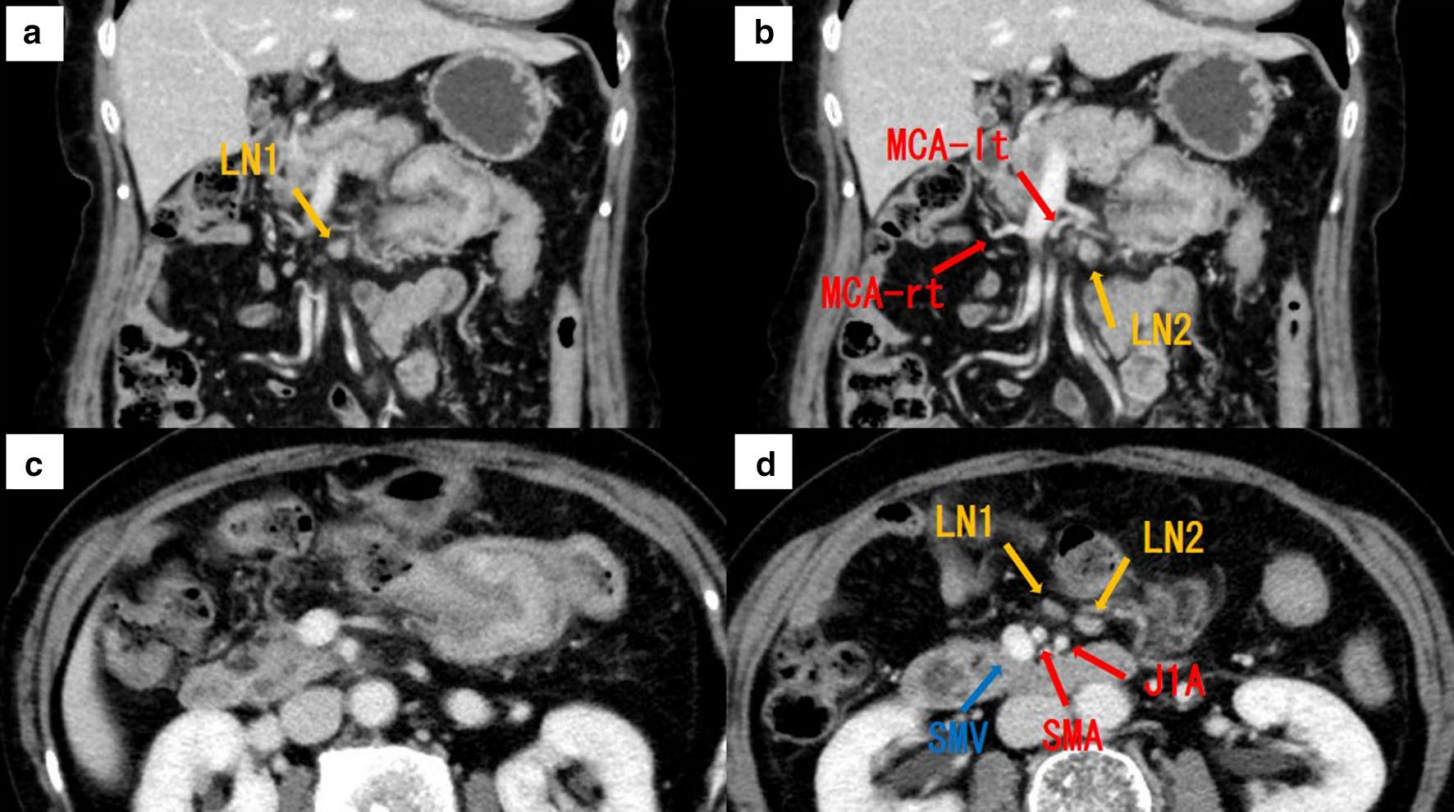
Abdominal contrast CT findings. **a** The tumor region (approximately 5 cm) with contrast effect was found to be invaginated into the anal intestinal tract on the left side of the transverse colon, but with no visible serosal invasion. **b** Swollen regional lymph nodes (approximately 10 mm) were found along the root and left branch of the middle colic artery. LN, lymph node; MCA-lt, left branch of middle colic artery; MCA-rt, right branch of middle colic artery; SMA, superior mesenteric artery; SMV, superior mesenteric vein; J1A, jejunal 1st artery

Subsequently, she presented to our institution with a sensation of pressure at the upper abdominal region as a chief complaint at the age of 76 years. On abdominal computed tomography (CT), a contrast-enhanced tumor lesion (approximately 5 cm) was invaginated into the anal side of the intestinal tract on the left side of the transverse colon (Fig. 3a–c), and swollen regional lymph nodes (approximately 10 mm) were located along the root and left branch of the middle colic artery (Fig. 3a, b, d). Ascites, peritoneal dissemination, and distant metastasis were not observed. Her blood chemistry showed no abnormal findings, including tumor marker levels, and she had carcinoembryonic antigen (CEA) levels of 0.8 ng/mL, and carbohydrate antigen 19-9 (CA19-9) levels of 3.7 U/mL.

**Fig. 4:**
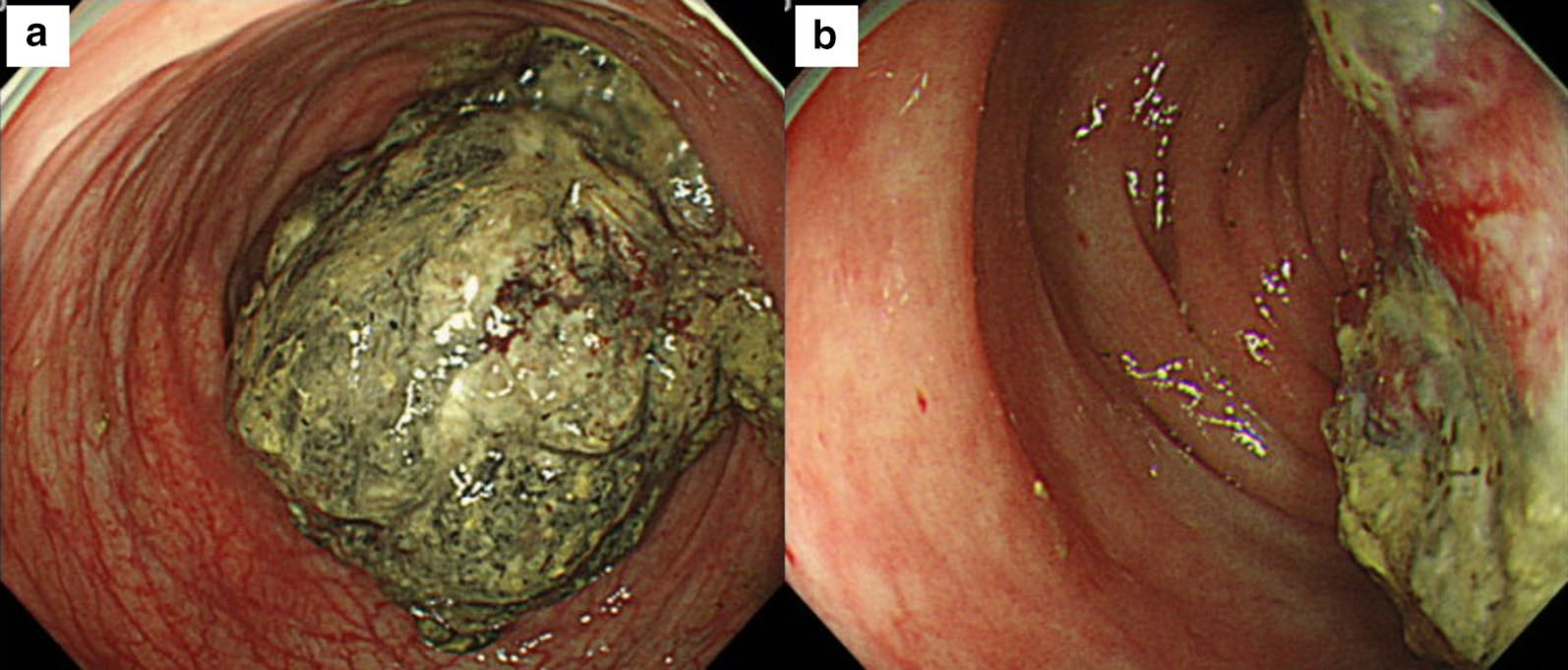
Colorectal endoscopy findings. **a** A torose lesion was found on the left side of the transverse colon (located 50 cm from the anal verge). **b** The tumor surface was covered with necrotic tissue and biopsy was performed from the exposed area

Colorectal endoscopy revealed a torose lesion on the left side of the transverse colon (50 cm from the anal verge), consistent with the site of the prior EMR (Fig. 4a). Its surface was covered in necrotic tissue, and subsequently biopsies the exposed area (Fig. 4b). Total colonoscopy was diligently performed, showing no polyps, other lesions, or scars from the prior EMR. Immunohistological examination showed that the spindle-shaped tumor cells were positive for α-SMA and negative for c-kit and CD34.

We diagnosed her with recurrence and enlargement of the leiomyoma that had been incompletely resected 3 years earlier based on the pathological similarities and occurrence site. (i.e., no other lesions had been observed during the previous EMR, no scarring due to the EMR or other lesions observed on the latest total colonoscopy, same distance from the anal verge as the prior lesion and same direction of the tumor base, and the lesions presented in the left transverse colon on coronal CT). We performed partial left transverse colectomy and lymph node dissection along the root and left branch of the middle colic artery.

**Fig. 5:**
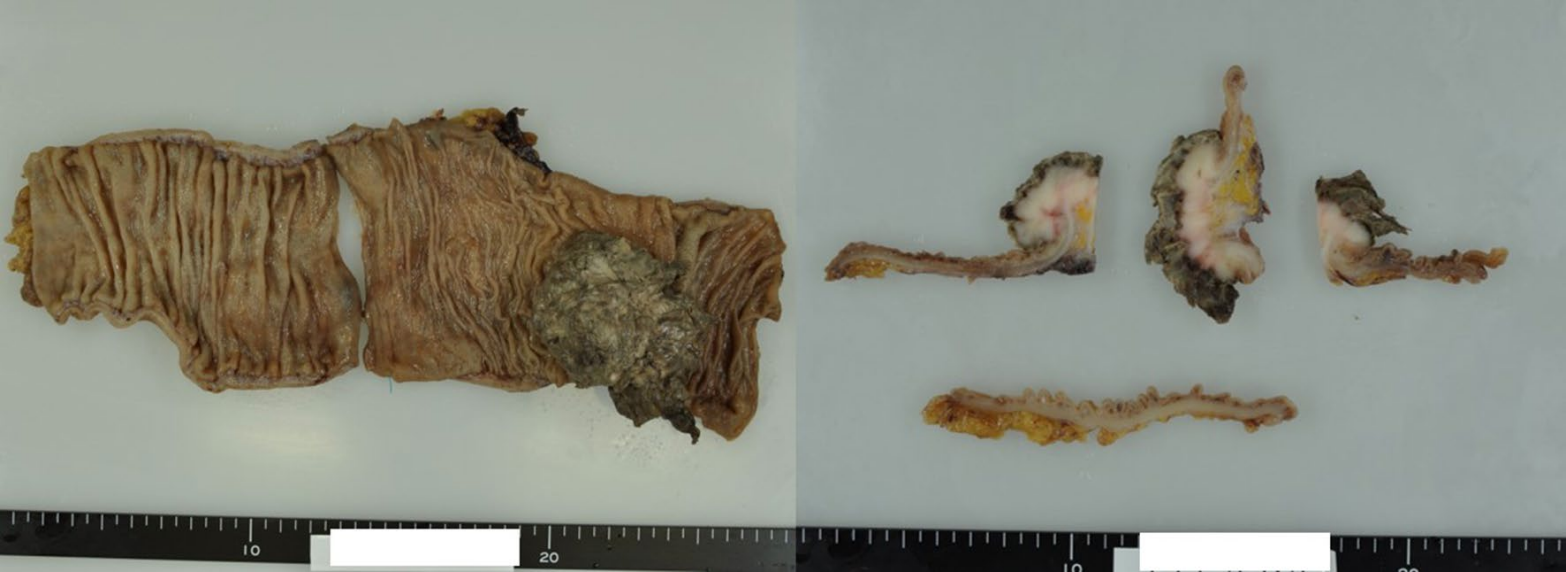
Characteristics of the resected specimen. Similar to a type1 tumor by the Borrmann classification (size: 55 × 50 mm) with necrosis on the surface and a milky solid component inside

**Fig. 6:**
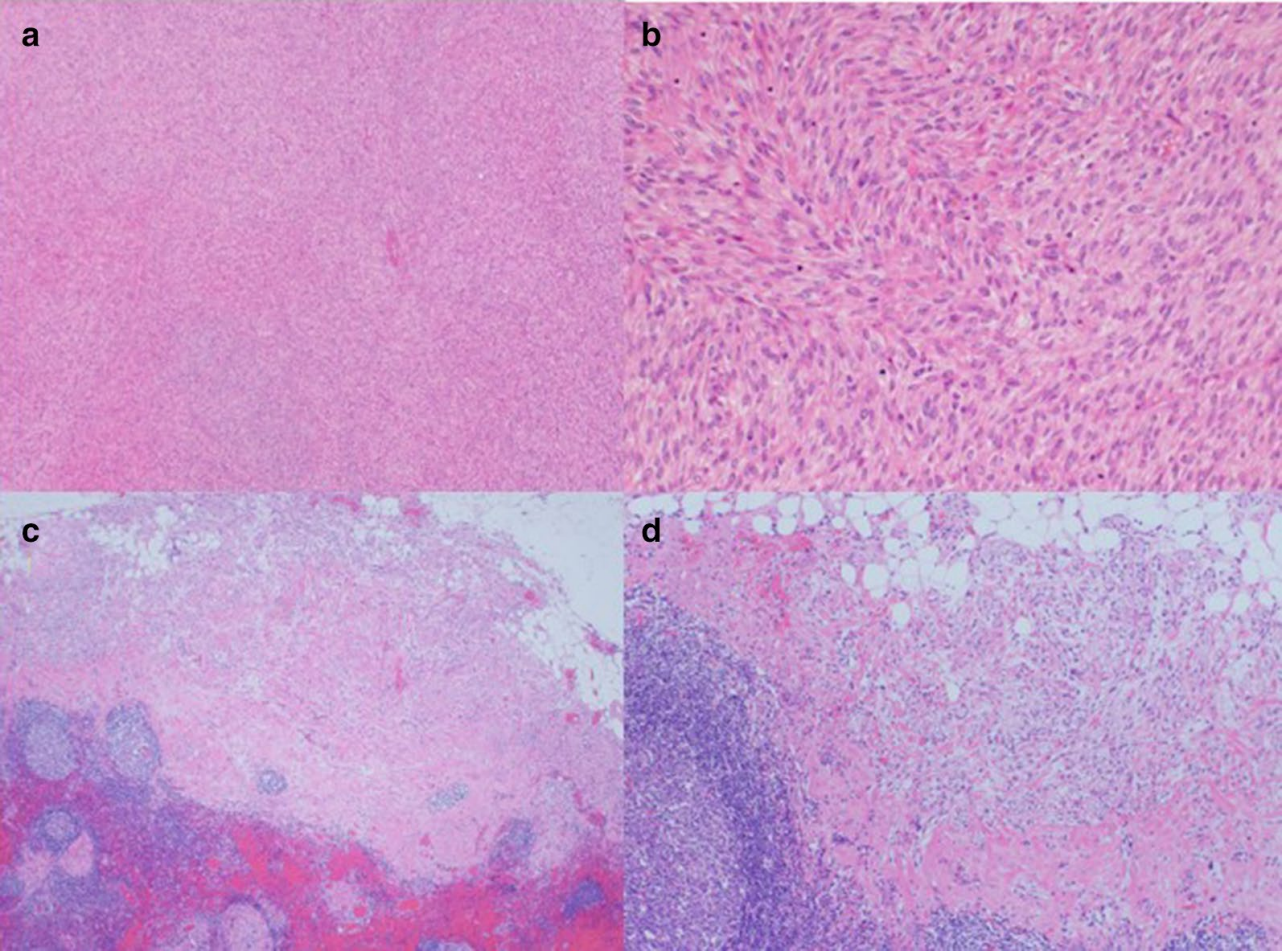
Microscopic findings of the tumor. **a** Hematoxylin–eosin staining (×40). **b** Hematoxylin–eosin staining (×100). These figures show numerous long spindle-shaped cells with relatively high cell density in the form of palisade, complex, or diffusion. Tumor cells maintain acidophilic and clear endoplasmic reticula, whereas some cells with mild-to-moderate nuclear grades with strange or giant form were scattered. The mitotic activity was 30–40/10 high-power field (HPF). **c** Hematoxylin–eosin staining (×40). **d** Hematoxylin–eosin staining (×100). These figures show metastasis of the dissected lymph nodes

**Fig. 7:**
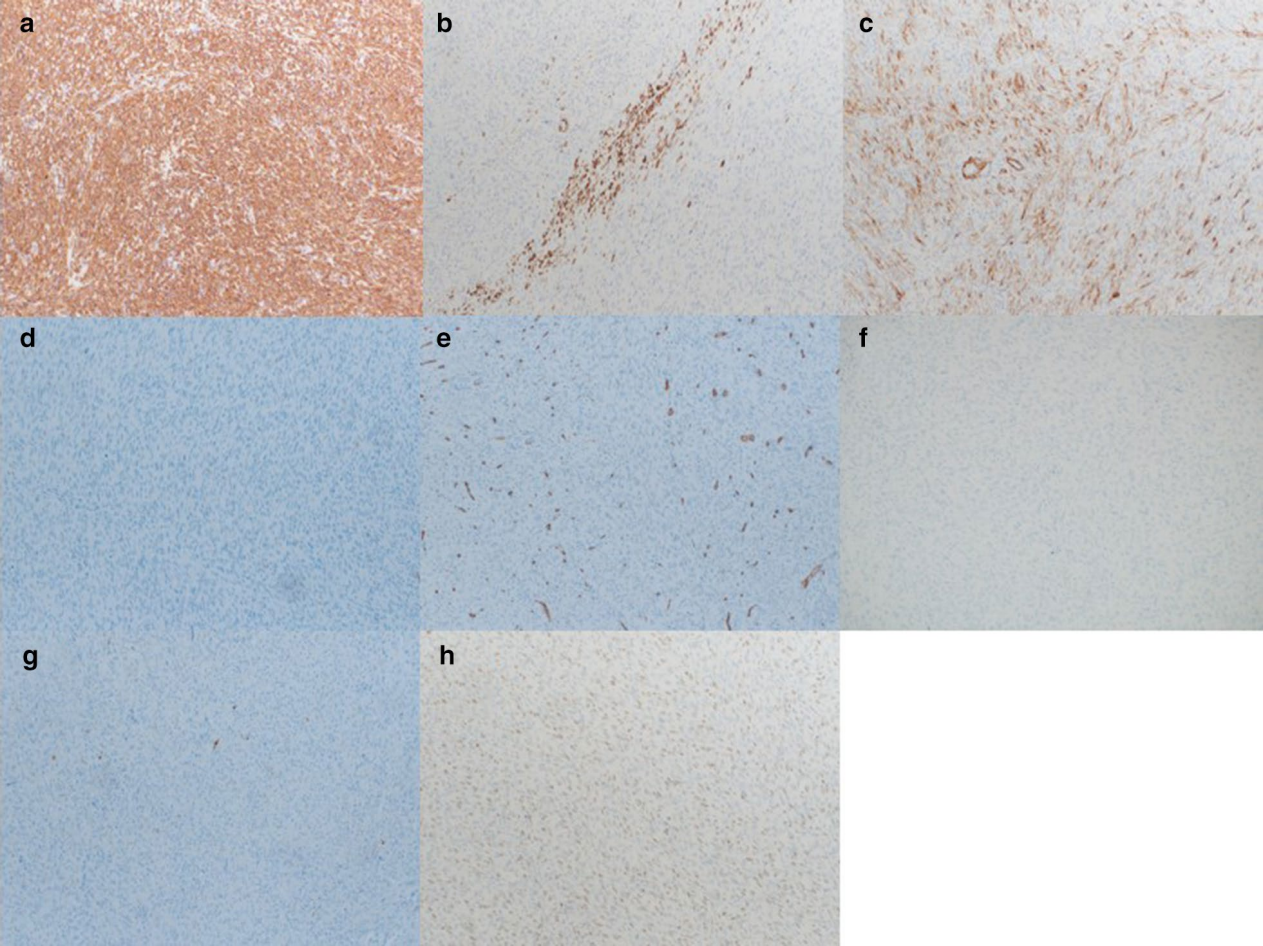
Immunohistological characteristics of the tumor. **a** Positive for α-smooth muscle actin (×100). **b** Positive for desmin (×100). **c**: Positive for h-caldesmon (×100). **d**: Negative for c-kit (×100). **e** Negative for CD34 (×100). **f** Negative for DOG-1 (×100). **g**: Negative for the S-100 protein (×100). **h** 70–80% for Ki-67 labeling index

Examination of the resected specimen showed the tumor to be a torose lesion (size: 55 × 50 mm) with necrosis on its surface and a milky solid component on the inside (Fig. 5). HE staining showed scattered long spindle-shaped cells with relatively high cell density and mild-to-moderate nuclear grades. The mitotic activity was 30–40/10 high-power field (HPF) (Fig. 6a, b). The tumor was mainly located in the proper muscular layer without exposure to the serous surface. Metastasis was found in 1 lymph node one dissected from the root of the middle colic artery from a total of 14 resected nodes. (Fig. 6c, d). Tumor cells were immunohistologically positive for α-SMA and h-caldesmon; partially positive for desmin; negative for c-kit, CD34, DOG-1, and the S-100 protein; and showed a Ki-67 labeling index of 70–80%. Similar findings were observed in the metastatic lymph node (Fig. 7). We performed an additional immunohistological examination of the initial leiomyoma specimen, which showed that it was positive for desmin and h-caldesmon, and several percent for Ki-67 labeling index (Fig. 2g–i).

Based on these results, the patient was diagnosed with leiomyosarcoma of the transverse colon with lymph node metastasis, which occurred due to malignant transformation of the incompletely resected leiomyoma. The TNM classification was T2bN1M0stage IIIb, according to the Union for International Cancer Control stage classification system (8th edition), and the histological grade was G3, according to the Federation Nationale des Centres de Lutte Contre le Cancer grading system. She did not receive adjuvant chemotherapy during the follow-up period, but has survived without recurrence for two years.

## Discussion

Smooth muscle tumors of soft tissues throughout the body can often be divided into two groups (leiomyoma and leiomyoma). Nuclear atypia, mitotic activity, and the presence/absence of coagulative necrosis are important parameters when classifying these tumors as benign or malignant [5]. Immunohistological diagnosis is very useful in the identification of gastrointestinal mesenchymal tumors, and as α-SMA, desmin, and h-caldesmon are positive in most cases of leiomyosarcoma, it is necessary to exclude GISTs and neurogenic tumors according to CD34 and the S-100 protein negativity, respectively [5].

Leiomyosarcoma generally has a poor prognosis; according to a report from Yamamoto et al. [6], the 5-year survival rate of 29 patients with gastrointestinal leiomyosarcoma was 51.6%, and the overall survival rate was significantly shorter in patients with tumors sized 5 cm or larger, but the primary organ and mitotic activity did not appear to affect prognosis. Furthermore, according to Miyajima et al.’ [7] study of 115 leiomyosarcoma patients (with no description of the primary organ), the 5-year survival rates according to the American Joint Committee on Cancer (AJCC; 5th edition) staging system were 78% for stage I, 49% for stage II, 35% for stage III, and 0% for stage IV. Surgical treatment, including extended resection, is the therapeutic measure of choice in according to the Guidelines of the European Society for Medical Oncology Cancer Congress for adult soft tissue tumors [8]. Neoadjuvant chemotherapy and postoperative radiation therapy can also be administered to improve radical resection outcomes in high-risk groups or allow for conversion to surgery [8]. On the other hand, chemotherapy can be offered for unresectable cases, but does not provide a satisfactory therapeutic effect [9]. Complete tumor resection is the only effective treatment for gastrointestinal leiomyosarcoma as well as other soft tissue tumors.

In soft tissue tumors, except for epithelioid sarcoma and clear cell sarcoma, lymph node metastasis is rare [8]. Anzai et al. and Yahagi et al. reviewed reports of 14 cases of leiomyosarcoma of the rectum and 27 cases of leiomyosarcoma of the colon published after 1998, when the diagnosis by immunostaining method was established [10, 11]. Although some cases of colorectal leiomyosarcoma have since been reported, only 1 case exhibited lymph node metastasis [6, 10]. The clinicopathological characteristics of this previous case and our own are summarized in Table 1 [6, 10]. Among these 2 cases, both patients were females in the age range of 72 to 76 years, and the primary organs were the rectosigmoid and left side of the transverse colon. In terms of macroscopic type and maximum diameter, the previous case showed an ulcerated mass of 110 mm, and ours had a pedunculated mass of 55 mm. The invasion depth ranged between T3 and T4b, demonstrating locally advanced disease in both cases. The number of metastatic lymph nodes was one in both cases. The mitotic activity was 30–40/10HPF to 10/HPF, and the Ki-67 labeling index was also high in both (70–80%). Based on these findings, it can be seen that the 2 cases had similar characteristics.

**Table 1:** Reported cases of colorectal leiomyosarcoma with lymph node metastasis

Case	Author/year	Age (years)/sex	Location	Diameter (mm)	Macroscopic form	Surgery	Invasion depth	Number of lymph node metastases	Mitotic activity	Ki-67 index (%)	Outcome (months)
1	Anzai [10]/2017	76/F	R	110	Torose lesion with ulcer	Rectal anterior resection	T4b (ovary)	1	10/HPF	80	Alive (60)
2	Our case	72/F	T	55	Type 1	Transverse colon resection	T3	1	30–40/10 HPF	70–80	Alive (18)

*M* male, *F* female, *R* rectum, *T* transverse colon, *HPF* high-power field

The efficacy of preventive lymph node dissection remains controversial, but as a lymph node with suspected metastasis was found on preoperative CT in our patient, colectomy with lymph node dissection was performed under the presumption of epithelial malignancy. Although there are only a small number of patients that exhibit lymph node metastasis because complete resection by surgery is the only radical treatment, we should select an adaptation case for dissection by taking advantage of preoperative imaging diagnosis as CT, positron emission tomography, and magnetic resonance imaging.

Smooth muscle tumors can occur in soft tissues throughout the body; for example, benign and malignant uterine smooth muscle tumors, which can be precisely classified by multiple methods [5, 12]. Rare cases of malignant uterine leiomyomas have been reported, such as benign metastasizing leiomyoma, malignant transformation to leiomyosarcoma, and malignant transformation of benign metastasizing leiomyoma [12–19]. According to several reports, malignant transformation of fibroids among uterine leiomyoma carriers may occur at an incidence rate of 0.23%, even in relatively small lesions [13, 15]. In addition, malignant transformation can reportedly occur in lesions with active mitotic activity, and that immunohistological analyses (e.g., p53, p16, and Ki67 staining) may be effective for its identification [12, 13]. On the other hand, to the best of our knowledge, there are no reports on transformation of colorectal leiomyoma to leiomyosarcoma. Miettinenn et al. [5] classified smooth muscle tumors of the gastrointestinal tract into the following three categories: intramural leiomyoma, leiomyoma of the muscularis mucosa, and leiomyosarcoma. Although recurrence often occurs upon incomplete resection of a gastrointestinal leiomyoma, it is exceptionally rare to encounter an atypical lesion that transforms to a malignancy because of the generally routine complete resection and careful follow-up for gastrointestinal leiomyoma [5].

Recurrence and malignant transformation after gastrointestinal leiomyoma, as in our case, may be caused by the initial incomplete resection and the lack of postoperative follow-up.

## Conclusion

We have reported a case of leiomyosarcoma of the transverse colon with lymph node metastasis that was malignantly transformed from a leiomyoma. We should select patients that would benefit from lymph node dissection by preoperative imaging diagnosis, keeping in mind the possibility of colorectal leiomyosarcoma with lymph node metastasis. Complete resection and postoperative follow-up according to tumor size and AJCC stage is critical considering the malignant potential of colorectal leiomyosarcoma.

## Data Availability

All data analyzed in this report are included within the article.

## Abbreviations

GIST: Gastrointestinal stromal tumor
EMR: Endoscopic mucosal resection
HE: Hematoxylin and eosin
α-SMA: α-Smooth muscle actin
CT: Computed tomography
CEA: Carcinoembryonic antigen
CA19-9: Carbohydrate antigen 19-9
HPF: High-power field
AJCC: American Joint Committee on Cancer

## References

[1] Hirota S, Isozaki K, Moriyama Y, Hashimoto K, Nishida T, Ishiguro S (1998). Gain-of-function mutations of c-kit in human gastrointestinal stromal tumors. Science.

[2] DeMatteo RP, Lewis JJ, Leung D, Mudan SS, Woodruff JM, Brennan MF (2000). Two hundred gastrointestinal stromal tumors: recurrence patterns and prognostic factors for survival. Ann Surg.

[3] Miettinen M, Furlong M, Sarlomo-Rikala M, Burke A, Sobin LH, Lasota J (2001). Gastrointestinal stromal tumors, intramural leiomyomas, and leiomyosarcomas in the rectum and anus: a clinicopathologic, immunohistochemical, and molecular genetic study of 144 cases. Am J Surg Pathol.

[4] Miettinen M, Sarlomo-Rikala M, Sobin LH, Lasota J (2000). Gastrointestinal stromal tumors and leiomyosarcomas in the colon: a clinicopathologic, immunohistochemical, and molecular genetic study of 44 cases. Am J Surg Pathol.

[5] Miettinen M, Fetsch JF (2006). Evaluation of biological potential of smooth muscle tumours. Histopathology.

[6] Yamamoto H, Handa M, Tobo T, Setsu N, Fujita K, Oshiro Y (2013). Clinicopathological features of primary leiomyosarcoma of the gastrointestinal tract following recognition of gastrointestinal stromal tumours. Histopathology.

[7] Miyajima K, Oda Y, Oshiro Y, Tamiya S, Kinukawa N, Masuda K (2002). Clinicopathological prognostic factors in soft tissue leiomyosarcoma: a multivariate analysis. Histopathology.

[8] Casali PG, Abecassis N, Aro HT, Bauer S, Biagini R, Bielack S (2018). Soft tissue and visceral sarcomas: ESMO-EURACAN Clinical Practice Guidelines for diagnosis, treatment and follow-up. Ann Oncol.

[9] Maki RG, Wathen JK, Patel SR, Priebat DA, Okuno SH, Samuels B (2007). Randomized phase II study of gemcitabine and docetaxel compared with gemcitabine alone in patients with metastatic soft tissue sarcomas: results of sarcoma alliance for research through collaboration study 002 [corrected]. J Clin Oncol.

[10] Anzai H, Nozawa H, Tanaka J, Yasuda K, Otani K, Nishikawa T (2017). Giant leiomyosarcoma of the rectum with lymph node metastasis: a case report and review of the literature. Int J Surg Case Rep.

[11] Yahagi M, Ishii Y, Hara A, Watanabe M (2019). Laparoscopic surgery to treat leiomyosarcomas of the sigmoid colon: a case report and literature review. Surg Case Rep.

[12] Kim JH, Choi YJ, Kim DC, Lee SJ (2010). Leiomyosarcoma arising in a patient with prior mitotically active leiomyoma. J Obstet Gynaecol Res.

[13] Yanai H, Wani Y, Notohara K, Takada S, Yoshino T (2010). Uterine leiomyosarcoma arising in leiomyoma: clinicopathological study of four cases and literature review. Pathol Int.

[14] Di Luigi G, D'Alfonso A, Patacchiola F, Di Stefano L, Palermo P, Carta G (2015). Leiomyosarcoma: a rare malignant transformation of a uterine leiomyoma. Eur J Gynaecol Oncol.

[15] Bharambe BM, Deshpande KA, Surase SG, Ajmera AP (2014). Malignant transformation of leiomyoma of uterus to leiomyosarcoma with metastasis to ovary. J Obstet Gynaecol India.

[16] Al Ansari AA, Al Hail FA, Abboud E (2012). Malignant transformation of uterine leiomyoma. Qatar Med J.

[17] Raposo MI, Meireles C, Cardoso M, Ormonde M, Ramalho C, Pires M (2018). Benign metastasizing leiomyoma of the uterus: rare manifestation of a frequent pathology. Case Rep Obstet Gynecol.

[18] Jo HC, Baek JC (2018). Case of pulmonary benign metastasizing leiomyoma from synchronous uterine leiomyoma in a postmenopausal woman. Gynecol Oncol Rep.

[19] Song KS, Keum DY, Hwang IS (2017). Malignant transformation of pulmonary benign metastasizing leiomyoma. Korean J Thorac Cardiovasc Surg.

